# Short Term Effects of Football Headers on Reaction Time and Symptomology in Males and Females

**DOI:** 10.1002/ejsc.70251

**Published:** 2026-10-07

**Authors:** Yusef Mohammed, Rithik Rajan, Benjamin Tari, Flaminia Ronca

**Affiliations:** ^1^ Division of Surgery and Interventional Sciences Institute of Sport, Exercise and Health University College London London UK

**Keywords:** cognition, executive function, impact, SCAT, sub‐concussive

## Abstract

**Introduction:**

Football heading is a form of repetitive head impact linked to cognitive decline. Although the English Football‐Association have implemented heading restrictions, evidence guiding sex‐specific protocols remains limited. This study explored the acute effects of a standardized heading drill on reaction time (RT) and concussion symptomology and whether sex moderates these outcomes.

**Methods:**

51 participants (25 females; mean age 22.8 ± 4.1 years), comprising recreational and university‐level footballers, were non‐randomly assigned to a heading group performing a 10‐header drill, or a non‐heading control group completing a regulated rest period. Immediately before and after the interventions, both groups completed tasks assessing simple reaction time, sustained attention, inhibition, and spatial timing anticipation and self‐reported concussion symptoms via the Sport Concussion Assessment Tool, sixth edition (SCAT‐6).

**Results:**

SCAT scores increased significantly post‐intervention only in the heading group (*p* = 0.002, *dz* = 0.59), with higher post‐heading scores compared to the control group (*p* = 0.009, *d* = 0.66). No significant main effect of sex (*p* = 0.08) nor any interactions involving sex were found for RT and symptomology (*ps* ≥ 0.22). No significant changes in RT for any cognitive test were observed for any condition (*ps* ≥ 0.14).

**Conclusion:**

These findings suggest that a 10‐header‐drill has significant effects on symptomology, but no measurable effect on RT, and that sex did not influence either outcome. This supports the need to monitor sub‐concussive symptoms post‐heading. Future research should assess both immediate and longer‐term effects of stronger, game‐representative headers, across a wider range of cognitive domains.

## Highlights

- A standardized 10‐header drill significantly increased self‐reported concussion‐like symptoms, with headache, head pressure, dizziness, and confusion driving this effect.
- Reaction time, error rates, and spatial timing anticipation showed no significant change following heading, suggesting that acute cognitive performance may be preserved even as subjective symptom burden rises after sub‐concussive impacts.
- Sex did not moderate the effects of heading on symptomology or cognitive performance, although these analyses were exploratory.
- The findings support routine symptom monitoring following heading exposure, while adequately powered studies are needed to determine whether responses differ by sex.

## 1 Introduction

Football is the most popular sport worldwide with nearly 250 million people participating (Encyclopedia Britannica [Internet] 1999). Football is unique in that it is the only contact sport in which participants utilise the head to advance play (Niedfeldt 2011). Heading is classified as a voluntary sub‐concussive impact which, in isolation, does not induce noticeable concussive symptoms (McCrory et al. 2017). However, repetitive head impacts (RHI) are highlighted as a risk factor for the development of neurodegenerative diseases such as Alzheimer's disease and chronic traumatic encephalopathy (Baugh et al. 2012; Nowinski et al. 2022). Growing awareness of potential long‐term consequences of RHIs on cognition has led the England Football Association (FA) to advise a maximum of 10 higher‐force headers per week (Premier League [Internet] 2021) at professional levels, and to prohibit heading at and below the under‐12 level (E and ngland Football [Internet] 2023). The FA have also recommended that clubs create player profiles, which include sex and other individual‐specific variables, to better guide training and play (Premier League [Internet] 2021). Despite such recommendations, there is still a dearth of research examining the effects of headers on cognitive performance, and whether sex might further influence these outcomes. A better understanding of such effects would support the designation of evidence‐based guidelines and enable the implementation of sex‐specific or player‐tailored training recommendations.

Notably, the effects of acute heading on cognitive function remains inconclusive and is likely due to methodological differences across studies. Previous works have utilised the pro‐and antipoint tasks to investigate simple reaction time and inhibitory control, respectively, following headers (Balagopal et al. 2020; Shamloo et al. 2023; Koerte et al. 2017; Zhang et al. 2013). These and other studies (Kontos et al. 2011; Kern et al. 2024), (Rieder and Jansen 2011) reported either no statistically significant changes or shortened (i.e., improved) reaction times (RTs) following heading. Some authors have acknowledged that headers were performed in the context of physical activity which could have been the main driver of shortened RTs after heading. Exercise levels were not often controlled for and this constitutes a significant confounding factor due to the oft‐reported benefits of exercise to cognitive function (Byun et al. 2014).

Previous research has frequently relied on observational training data, and few have utilized standardized heading protocols, thus leading to inconsistencies in heading frequency and intensity. However, Di Virgilio and colleagues included a heading procedure, where participants completed 20 headers from a distance of 6 m (Di Virgilio et al. 2016). The authors reported no significant effect on RT, but found a 67% increased error rate on a paired associative learning task, as well as a prolonged corticomotor inhibition silent period. Similarly, Huber and colleagues assessed whether junior footballers' neurophysiological reflexes were influenced by a series of 10 headers (Huber et al. 2023). Participants were assessed via the King‐Devick test (i.e., a measure of saccade speed), and their pupillary light reflexes and visual‐evoked potentials were also recorded. Results demonstrated no significant differences between pre‐ and post‐heading on these measures. In contrast to these null‐results, Ashton, and Parr and colleagues found a significant reduction in King‐Devick test performance when participants completed 20 headers (Ashton et al. 2021; Parr et al. 2023). Accordingly, these mixed results demonstrate the potential domain‐ and dose‐specific differences to cognitive outcomes.

Research investigating sex differences in the effects of heading has shown that female footballers experience 32% greater head accelerations than males after heading a standard football (Caccese et al. 2018; Bretzin et al. 2017). Heading exposure has also been shown to alter brain structure in the long‐term. Both Koerte, and Lipton and colleagues have reported former professional footballers experience cortical thinning and white matter microstructure abnormality in the temporo‐occipital region, respectively (Koerte et al. 2022; Lipton et al. 2013). Retired female footballers were also found to present with more pronounced changes to white matter microstructure than their male counterparts, despite comparable header exposure (Rubin et al. 2018). These results highlight evidence for the potential for greater heading‐related cognitive detriments for females in the long‐term. Sex differences have also been observed in concussion incidence and outcomes with females shown to have a higher risk of concussion and to experience worsened symptomology (Dick 2009) compared to males (i.e., longer RT, impaired memory and visual motor speed (Chiang et al. 2009) and visual memory (Covassin et al. 2007)).

However, data from the NCAA‐DoD Concussion Assessment, Research and Education (CARE) Consortium indicate that when male and female athletes in comparable collegiate sports have equivalent access to sports medicine provision, concussion incidence and recovery trajectories are broadly similar, with females differing mainly in acute symptom burden and vestibular‐ocular findings (Caccese et al. 2024). Reported sex differences may therefore reflect modifiable extrinsic factors, such as access to care and symptom‐reporting behaviour, as much as intrinsic biological differences.

To date, only two studies, by Kontos et al., and Rieder and Jansen, have investigated the acute effects of headers on RT between sexes (Kontos et al. 2011; Rieder and Jansen 2011). Both reported no sex differences; however, confounding variables such as exercise (see above) were not controlled for. Rieder and Jansen also reported that females experienced more frequent headaches than males post‐heading, suggesting the possibility for sex‐related differences in experienced symptomology (Rieder and Jansen 2011). Accordingly, the aim of this study was to investigate the immediate effects of heading on cognitive performance and symptomology following a standardized heading drill, and associated sex differences. We hypothesised that cognition and symptom severity would worsen following heading, and females would exhibit greater effects than males.

## 2 Methods

### 2.1 Participants

Participants were recruited via convenience and snowball sampling and were non‐randomly allocated into two groups, a heading group and a non‐heading control group; based on availability to attend the testing sessions. Participants were a mixture of recreational and university‐level football players. Participants were excluded by age (≤ 18 and ≥ 40 years old), any existing or known neurological conditions, or any incidence of concussion within 12 months. An a priori power analysis using G*Power indicated that a minimum sample of 34 participants (17 per group) was required to detect a medium effect of heading on RT (*f* = 0.25) with 80% power at *α* = 0.05. The final sample of 51 participants exceeded this requirement (Kern et al. 2024). All participants provided informed written consent of a protocol approved by the University College London Research Ethics Committee (13985.007) prior to undertaking any study activity. These activities aligned with the most recent iteration of the Declaration of Helsinki.

### 2.2 Study Design

Participants were instructed to attend and complete two sessions over the course of two separate days. Session 1 was not included in any analysis and served as a baseline familiarisation session to mitigate learning effects observed in pilot testing and literature (Parr et al. 2023). In the sessions, participants in the heading group performed cognitive tests prior to and immediately after the heading protocol (see below), whereas participants in the control group were instructed to complete the cognitive tests 5 minutes apart to standardise the time interval in both groups.

### 2.3 Heading Protocol

The heading drill required participants to header a football thrown to them from a distance of 5 m. Participants completed two practice headers using a size 5 football pumped to 8.5 PSI, which was in line with FA regulations (8.5–15.6 PSI) (Shewchenko et al. 2005) to ensure standardisation with normal training conditions. They then performed 10 headers using the same regulation football. This protocol was derived from the FA's recommendation of no more than 10 higher‐force headers per week (Premier League [Internet] 2021) and the 5 m distance was chosen to minimise high force headers whilst also attempting to be consistent with matches/training. Participants were instructed to head the ball as far as possible over the researcher's head in an effort to standardise exertion level.

Throws were delivered by one of four trained researchers, each familiarized with the standardized protocol prior to data collection. Researchers were instructed to throw the ball overhead, directed at head height from point A to point B (Figure 1). Ball velocity was quantified from 60 fps video footage as the time taken for the ball to travel between cone 1 and cone 2 (Figure 1). Mean delivery velocity was comparable across researchers (grand mean = 5.41 m/s, SD = 0.32; researcher means = 5.18–5.63 m/s). Inter‐rater reliability was assessed using a two‐way mixed‐effects intraclass correlation model, which indicated a good agreement amongst researchers, *ICC*(3,k) = 0.76 (95% CI [0.36, 0.93]), *p* < 0.05 (Koo and Li 2016). Ball velocity was checked from the video footage for every participant rather than at the outset only, allowing drift across a testing session to be detected. The delivery method was selected with reference to the UEFA expert‐panel quality criteria for studies assessing the acute effects of heading (Peek et al. 2024), while the heading dose was informed by observed in‐training heading frequencies reported by Townsend et al. (Townsend et al. 2025).

**FIGURE 1:**
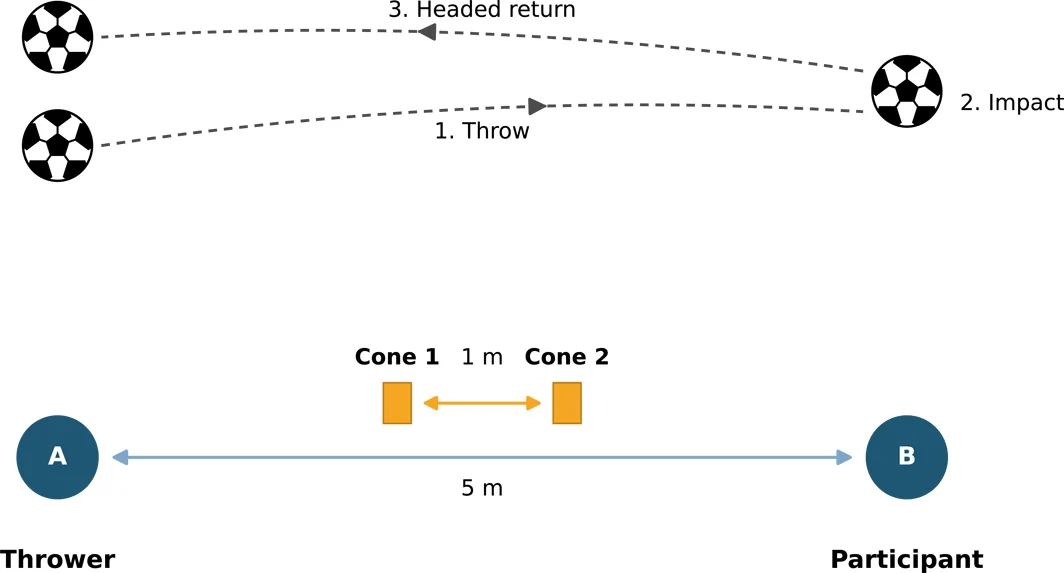
Structure of the controlled heading drill. A thrower was stationed at point A and a participant stood at point B 5 m away. Cones were placed at the 2 and 3 m were used to provide a reference point for video analyses and to measure ball speeds.

This heading protocol was also designed as an isolated drill, separate from play or training, with the cognitive testing area positioned adjacent to the drill setup to control for and minimise participant exertion and the stimulatory effects of exercise.

### 2.4 Cognitive Tests and Symptom Reporting

Prior to and immediately after the heading drill (for the heading group) or the 5‐min resting period (for the control group), participants completed a questionnaire, and a cognitive battery similar to that used by Ronca and colleagues via the Gorilla platform on a computer (Ronca et al. 2025). First, both heading and control group participants rated their perceived concussion‐related symptoms on a scale of 0–100 via a modified Sport Concussion Assessment Tool, sixth edition (SCAT‐6) symptom scale (Ruben 2023). The scale included items such as dizziness, headache, and increased head pressure (see Supporting Information S1: Table S1 and Figure S1). Then, participants complete the cognitive battery which consisted of four tasks: simple reaction time (SRT), sustained attention (SA), inhibition (INH), and spatial timing anticipation (STA) task. The SRT task required participants to press the spacebar as soon as a ‘smiley’ or ‘winky’ face appeared on their screen. This task consisted of 30 trials with intertrial intervals (ITIs) of 1000, 1500, or 2000 milliseconds (ms). Mean RT was then calculated by taking the mean of all correct responses, defined as responses when an image (i.e., a smiley or winky face icon) was visible, after removing responses faster than 125 ms. Commission errors were counted when the spacebar was pressed during ITIs. The SA task was structured as a No‐Go/Go task where participants were instructed to press the spacebar when a ‘winky’ face was projected (*n* = 10 trials), and to withhold their response when a ‘smiley’ face was projected (*n* = 20 trials) (i.e., 30 total trials; ITIs 2000, 3000, 4000 ms). Mean RT was calculated by taking the mean of all correct Go responses, defined as responses when the ‘winky’ face was visible, after removing responses faster than 125 ms. Commission errors were characterised as responses when the ‘smiley’ face was visible. Regarding the INH task (i.e., the Go/No‐Go task), participants were required to press the spacebar when the ‘smiley’ faces were presented (*n* = 62 trials) and withhold responses when the ‘winky’ faces were presented (*n* = 12 trials) (i.e., 74 total trials; 300 ms ITI). Mean RT was then calculated by taking the mean of all correct Go responses, defined as responses when the ‘smiley’ face was visible, after removing responses faster than 125 ms. Commission errors were defined as responses when the winky face was visible. Finally, the STA task required participants to estimate the collision time between two footballs moving at 6–18 m/s along a horizontal line. The task involved 29 trials, with visible and obscured footballs, to simulate real sports timing demands. Timing error was defined as the absolute difference (ms) from the participants' response to the moment the footballs collided. All timing error values were transformed to absolute values prior to calculating mean scores. All stimuli were presented in a fixed pseudorandomised order. The tasks of the cognitive battery, their design and validation are presented in more detail by Ronca and colleagues (Ronca et al. 2025).

An exploratory factor analysis (principal axis factoring with varimax rotation) was conducted on the cognitive outputs from the smiley task battery (RTs for the SRT, SA, INH tasks, inter‐trial presses for the SRT task and commission errors for the SA and INH tasks). A two‐factor solution emerged, accounting for 45.5% of the total variance. The first factor (27.2% variance) showed strong loadings for the SRT (0.68), SA (0.66), and INH task (0.83) RTs, and was labelled Composite Reaction Time. The second factor (18.3% variance) loaded most strongly on INH commission errors (0.92) and was labelled Composite Error. Regression‐based factor scores were saved and used as dependent variables in subsequent analyses. Timing error (ms) from the STA task was analysed separately.

### 2.5 Statistical Analysis

All statistical analyses were conducted using RStudio (version 4.3.2). The factor analysis‐derived Composite Reaction Time and Composite Error were used to assess changes in the attention and inhibition battery (SRT, SA, INH tasks), while mean timing error (ms) was used to assess changes in STA task performance. Total SCAT (sum of all symptom severity scores) was used for preliminary generalised analyses before symptom evaluation. Higher values indicated worse symptomology. Data normality was assessed using histograms and Shapiro‐Wilk tests, with non‐normal variables (Composite Reaction Time, Composite Error, and STA timing error) transformed using a Box‐Cox procedure (SCAT symptom scores were not transformed). Independent variables were analysed via linear mixed‐effects models comprising a 2 (group: heading vs. control) × 2 (time: pre‐vs. post‐) fixed‐effects structure with a random intercept for participant. Fixed effects were evaluated with Type III tests using Kenward‐Roger approximated degrees of freedom. Because the within‐participant factor (time) had only two levels, sphericity assumptions did not apply. Significant interactions were decomposed using estimated marginal means with Bonferroni‐corrected pairwise contrasts, and effect sizes are reported as partial eta‐squared (ηp^2^) for omnibus effects and Cohen's dz for within‐participant contrasts. Sex differences were assessed using an analogous mixed‐effects model in which group was replaced by sex. These sex analyses were exploratory as the study was powered for the primary group × time comparison, not for sex effects. A sensitivity analysis indicated that the achieved subgroup sizes provided 80% power to detect only large sex differences. Regression analyses were performed to investigate whether changes in SCAT scores predicted changes in cognitive variables. Regression assumptions (linearity, homoscedasticity, and normality of residuals) were inspected via residual plots, and model fit is reported as adjusted *R* ^2^. Between‐group contrasts derived from the mixed‐effects models are reported with non‐integer denominator degrees of freedom; these reflect the Kenward‐Roger approximation applied to the mixed model rather than a Welch correction. Homogeneity of variance was assessed with Levene's test. Post‐intervention SCAT variance was substantially greater in the heading group than in the control group, so the equal‐variance assumption was not imposed on the between‐group contrast. The 15 individual SCAT‐6 symptom items were treated as a single family of exploratory paired contrasts: uncorrected *p*‐values are reported alongside Benjamini‐Hochberg false‐discovery‐rate‐adjusted values (Table 1), and item‐level effects are interpreted only where they survive adjustment. A significance level of *p* = 0.05 was used for all tests.

**TABLE 1:** Symptom scores (mean ± SD) for each of the individual SCAT items before and after the intervention in the Heading and Control groups.

	Heading group					Control group		
	Before	After	*p*‐value	*dz*	*pFDR*	Before	After	*p*‐value
Headache	11.9 (20.8)	24.8 (30.8)	0.005**	0.52	0.041	3.8 (7.7)	3.5 (6.5)	0.664
Head pressure	8.2 (18.4)	24.3 (29.2)	< 0.001***	0.67	0.008	1.6 (4.3)	2.6 (4.6)	0.223
Dizzy	8.7 (18.5)	18.5 (25.3)	0.013*	0.46	0.049	1.2 (2.9)	1.9 (4.0)	0.394
Balance	3.9 (9.7)	8.2 (17.1)	0.089	0.31	0.159	1.6 (3.5)	1.9 (3.6)	0.579
Fog	7.1 (15.7)	14.0 (25.8)	0.092	0.30	0.159	2.7 (5.2)	3.4 (4.8)	0.605
Drowsy	9.2 (19.6)	14.6 (24.7)	0.095	0.30	0.159	3.3 (6.6)	3.3 (6.3)	> 0.999
Sensitive to light	5.1 (15.1)	9.9 (21.8)	0.134	0.27	0.201	2.1 (5.1)	1.3 (3.0)	0.448
Blurred vision	2.1 (8.2)	4.9 (14.6)	0.212	0.22	0.289	1.1 (2.9)	2.3 (5.1)	0.333
Nervous	6.0 (15.4)	8.2 (17.2)	0.368	0.16	0.460	3.2 (5.8)	3.3 (5.6)	0.939
Irritable	5.9 (15.0)	7.3 (19.8)	0.596	0.09	0.687	2.6 (5.9)	1.0 (2.1)	0.120
Neck pain	8.3 (19.0)	8.1 (17.5)	0.849	−0.03	0.849	5.4 (11.8)	3.5 (8.8)	0.284
More emotional	5.7 (14.9)	6.3 (16.2)	0.755	0.05	0.809	4.2 (14.8)	3.5 (11.8)	0.445
Confusion	6.8 (16.2)	16.1 (25.5)	0.013*	0.46	0.049	3.0 (7.3)	2.7 (4.4)	0.865
Not feel right	6.3 (13.5)	14.5 (24.0)	0.023*	0.41	0.070	2.2 (4.7)	2.1 (5.3)	0.948
Slowed down	7.2 (13.9)	17.6 (27.3)	0.032*	0.39	0.081	4.4 (7.6)	5.4 (9.7)	0.510
Total SCAT‐6	102.3 (197.3)	197.4 (284.3)	0.009**	0.48	—	42.4 (41.7)	41.9 (41.7)	0.930

*Note: p*‐values indicate within‐group comparisons (paired tests). Significant differences are marked as *p < 0.05, **p < 0.01, ***p < 0.001. dz = Cohen's dz for the within‐participant pre‐post contrast in the heading group. pFDR = Benjamini‐Hochberg false‐discovery‐rate‐adjusted *p*‐value, computed across the family of 15 individual symptom items; the total SCAT‐6 score is the primary outcome and is not included in that family. Dizziness and confusion adjust to pFDR = 0.0500 and therefore fall only marginally below the 0.05 criterion. No control‐group contrast reached significance (all ps ≥ 0.120), so no adjustment is reported for that group.

## 3 Results

### 3.1 Participants

A total of 66 participants were recruited for the study. Fifteen participants withdrew due to time constraints, leaving 51 individuals (aged 18–36 years) in the final analysis. Participants were allocated to a heading group (*n* = 33; female = 17) and a non‐heading control group (*n* = 18; female = 8). The two groups were comparable in age, sex distribution, self‐reported weekly heading exposure and main sport (Table 2).

**TABLE 2:** Participant demographics and group characteristics (*N* = 51).

Variable	Total sample (*N* = 51)	Header group (*N* = 33)	Control group (*N* = 18)
Age (years), mean (SD)	22.8 (4.1)	23.0 (4.20)	22.3 (3.76)
Female sex, n (%)	25 (49)	17 (52)	8 (44)
Headers per week, mean (SD)	0.70 (0.8)	0.76 (0.9)	0.61 (0.6)
Main sport: Football, n (%)	29 (57)	19 (58)	10 (56)

*Note:* Percentages are based on the total *N* of each group unless otherwise indicated. Sex is reported as the number and percentage of female participants; male counts are the remainder.

### 3.2 Heading Impact on Composite Reaction Time and Composite Error

Results for the Composite Reaction Time and Composite Error indicated no significant main effects of time (*Fs*(1, 47) = 0.32 and 2.24, *ps* = 0.574 and 0.141, ηp^2^ = 0.01 and 0.01), group (*Fs*(1, 47) = 0.38 and 0.20, *ps* = 0.541 and 0.657, ηp^2^ = 0.01 and 0.002), or their interaction (*Fs*(1, 47) = 0.002 and 0.02, *ps* = 0.965 and 0.888, ηp^2^ = 0.001). Moreover, neither variable demonstrated a main effect of sex (*Fs*(1, 47) = 3.18 and 2.67, *ps* = 0.081 and 0.109, ηp^2^ = 0.06 and 0.05), nor were there any significant interactions involving sex, group, or time (*Fs*(1, 47) < 1.82, *ps* > 0.184, ηp^2^ < 0.04) (Figure 2).

**FIGURE 2:**
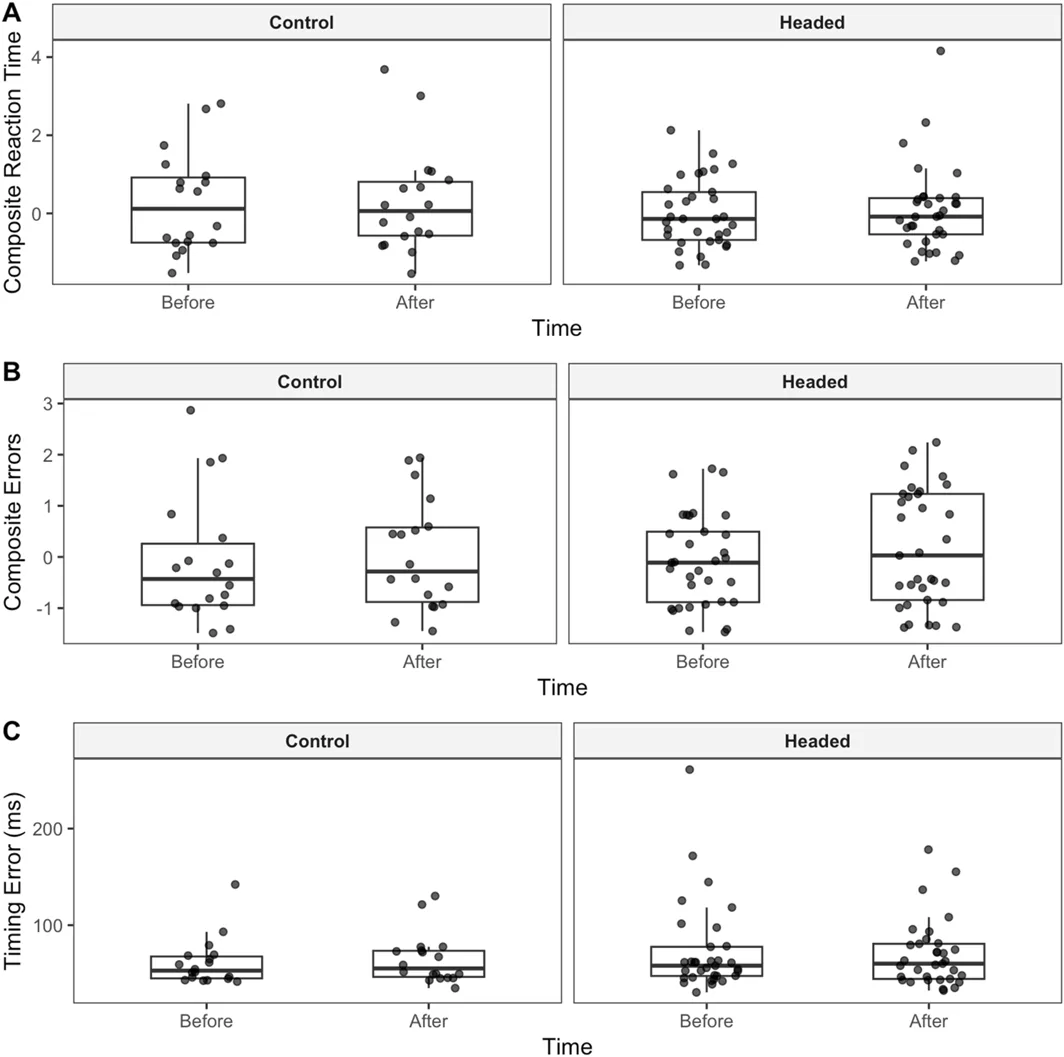
Composite scores derived from the factor analysis conducted on the cognitive testing battery, A = Composite Reaction Time (combines all the four reaction time variables calculated in milliseconds), B = Composite Error (combines all the total commission errors), C = Spatial Timing Anticipation (STA) task (timing error in milliseconds). Higher scores are indicative of worse performance on all tasks. No significant differences were observed between the control and heading group and there were no sex differences within the heading group.

Results for the STA task (Mean Positive Difference) revealed no significant main effects of time, (*F*(1, 47) = 0.10, *p* = 0.753, ηp^2^ = 0.002); group, (*F*(1, 47) = 0.13, *p* = 0.720, ηp^2^ = 0.003); or sex, (*F*(1, 47) = 0.60, *p* = 0.442, ηp^2^ = 0.01). The group × time interaction was also not significant, (*F*(1, 47) = 0.27, *p* = 0.606, ηp^2^ = 0.006) indicating that changes in STA performance over time did not differ between the heading and control groups. No significant two‐way or three‐way interactions involving sex were observed.

### 3.3 Symptom Analysis

In terms of total SCAT‐6 score, the analysis revealed a significant main effect of group, (*F*(1, 47) = 4.09, *p* = 0.049, *η*_*p*_^2^ = 0.08). The main effect of time, (*F*(1, 47) = 3.92, *p* = 0.054, *η*_*p*_^2^ = 0.08), and the group × time interaction, (*F*(1, 47) = 4.00, *p* = 0.051, *η*_*p*_^2^ = 0.08), both fell marginally above the conventional threshold. Given the a priori directional hypothesis and the fact that the interaction carried an effect size equivalent to the significant main effect of group (*η*_*p*_^2^ = 0.08), the interaction further analysed. Post‐hoc analysis indicated that participants in the heading group reported increased symptom scores after the heading drill (*t*(47) = 3.36, *p* = 0.002, dz = 0.59), while no significant change was observed in the control group (*t*(47) = 0.01, *p* = 0.992, dz < 0.001). Furthermore, post‐heading SCAT scores were significantly higher in the heading group compared to the control group (*t*(66.5) = 2.68, *p* = 0.009, *d* = 0.66, 95% CI [0.16, 1.15]) (Figure 3). The main effect of sex was not significant (*F*(1, 47) = 3.19, *p* = 0.081, ηp^2^ = 0.06) and no interactions involving sex, group, or time were observed (*Fs*(1,47) < 1.56, *ps* > 0.218, ηp^2^ < 0.03).

**FIGURE 3:**
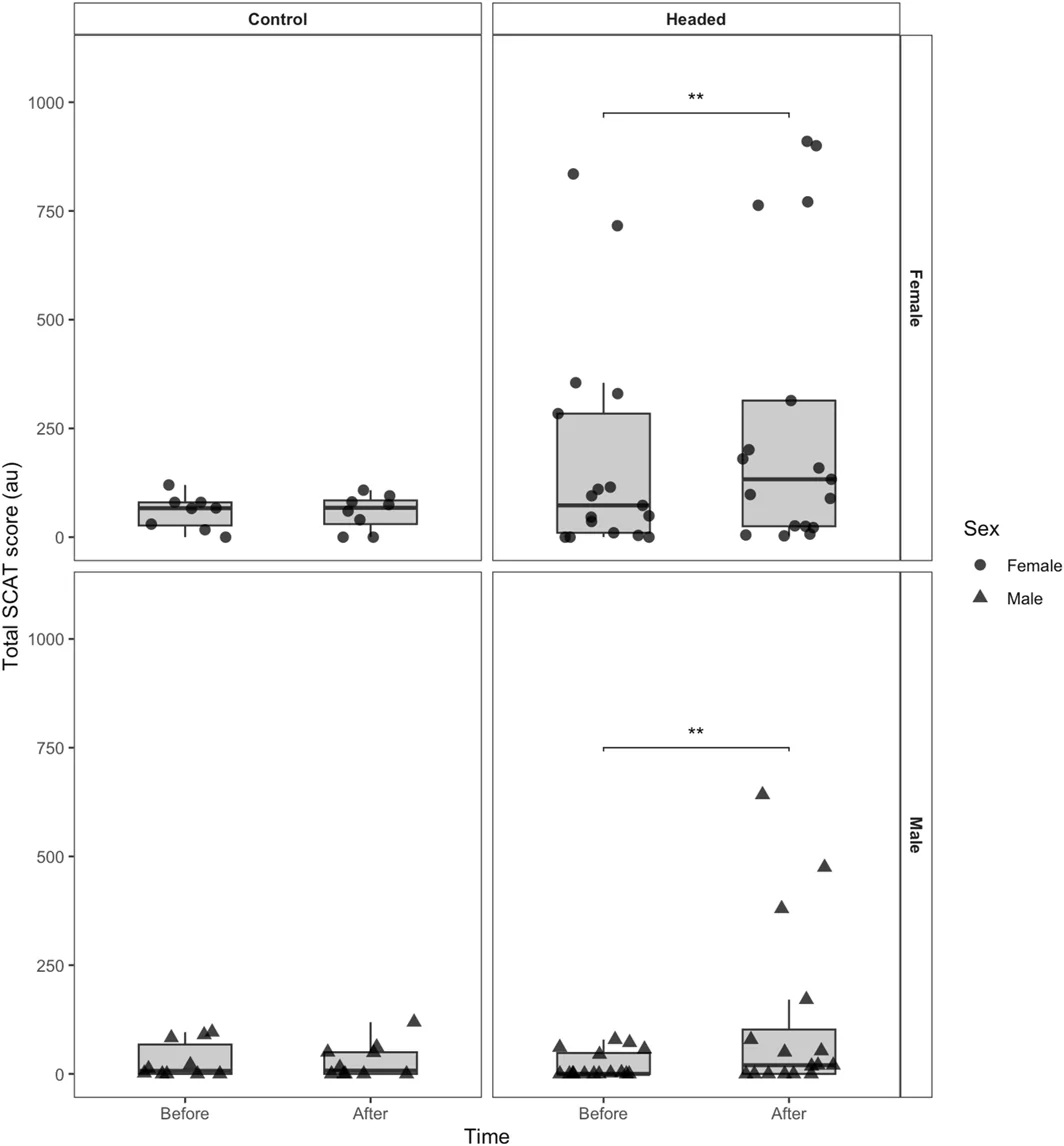
Boxplots of total SCAT‐6 symptom severity scores before and after the intervention in the heading group (*n* = 33) and control group (*n* = 18), stratified by sex. In the heading group, total SCAT scores increased significantly after heading (Pre: 102.3 ± 197.3; Post: 197.4 ± 284.3; paired *t*‐test, *p* = 0.009), whereas no change was observed in the control group (Pre: 42.4 ± 41.7; Post: 41.9 ± 41.7; *p* = 0.930). Post‐session estimated marginal mean comparisons from the linear mixed‐effects model revealed significantly higher SCAT scores in the heading group compared with controls (*t*(66.5) = 2.68, *p* = 0.009, *d* = 0.66). No significant sex differences in the magnitude of symptom change were found (*p* = 0.906 in the heading group; *p* = 0.953 in the control group). Data are mean ± SD. Asterisks denote the significance of the indicated comparison: **p < 0.01.

Individual items of the SCAT symptom questionnaire were also analysed separately (Table 1). In the heading group, significant increases in symptom severity were observed for headache (*t*(32) = 2.98, *p* = 0.005, *d*_*z*_ = 0.52); head pressure, (*t*(32) = 3.87, *p* < 0.001, *d*_*z*_ = 0.67); dizziness, (*t*(32) = 2.65, *p* = 0.013, *d*_*z*_ = 0.46); confusion, (*t*(32) = 2.62, *p* = 0.013, *d*_*z*_ = 0.46); not feel right, (*t*(32) = 2.38, *p* = 0.023, *d*_*z*_ = 0.41); and slowed down, (*t*(32) = 2.24, *p* = 0.032, *d*_*z*_ = 0.39). The total SCAT‐6 symptom score also increased significantly following the heading task, (*t*(32) = 2.78, *p* = 0.009, *d*_*z*_ = 0.49). No other symptoms showed significant pre–post differences within the heading group. In contrast, no significant changes were observed across any symptom domains in the control group (all *p*s > 0.05). Because the 15 symptom items constituted a family of related tests, *p*‐values were adjusted using the Benjamini‐Hochberg false‐discovery‐rate procedure (Table 1). Head pressure (pFDR = 0.008), headache (pFDR = 0.041), dizziness (pFDR = 0.049) and confusion (pFDR = 0.049) remained significant after adjustment, whereas the increases in not feeling right (pFDR = 0.070) and feeling slowed down (pFDR = 0.081) did not. Effect sizes for the individual items surviving correction ranged from small to medium (dz = 0.46–0.67).

### 3.4 Regression Analysis

Linear regression analyses were conducted within the heading group (*N* = 33) to examine if changes in SCAT‐6 scores predicted changes in performance, with sex interactions. For STA timing error, increases in SCAT‐6 scores were not significantly associated with changes in performance (*β* = −0.052, 95% CI [−0.125, 0.022], *t*(29) = −1.44, *p* = 0.161). Sex (*β* = 7.21, *p* = 0.354) and the SCAT‐6 by sex interaction (*β* = 0.047, *p* = 0.197) were also not significant. The overall model fit was *F*(3, 29) = 2.45, *p* = 0.083, Adjusted *R* ^2^ = 0.120. The model therefore accounted for approximately 12% of the variance in timing‐error change after adjustment for the number of predictors, although the model as a whole was not statistically significant (Table 3).

**TABLE 3:** Summary of results and interactions from the linear regression models.

Model A: Change in STA timing error (ms)			
Predictors	Estimates (B)	95% CI	*p*
(Intercept)	1.536	[−14.118, 17.190]	0.842
SCAT change	−0.052	[−0.125, 0.022]	0.161
Sex(Male)	7.207	[−8.447, 22.862]	0.354
SCAT change × Sex(Male)	0.047	[−0.026, 0.121]	0.197
Observations: 33			
*R* ^2^ = 0.202, adjusted *R* ^2^ = 0.120			
*F*(3, 29) = 2.45, *p* = 0.083			

**Model B: Change in composite error (au)**			
**Predictors**	**Estimates (B)**	**95% CI**	***p***
(Intercept)	0.171	[−0.188, 0.529]	0.338
SCAT change	0.0008	[−0.0008, 0.0025]	0.315
Sex(Male)	−0.117	[−0.475, 0.242]	0.511
SCAT change × Sex(Male)	0.0011	[−0.0006, 0.0027]	0.210
Observations: 33			
*R* ^2^ = 0.075, adjusted *R* ^2^ = −0.021			
Model fit: *F*(3, 29) = 0.78, *p* = 0.516			

Model C: Change in composite reaction time (au)			
**Predictors**	**Estimates (B)**	**95% CI**	***p***
(Intercept)	0.074	[−0.215, 0.363]	0.605
SCAT change	0.0001	[−0.0012, 0.0015]	0.841
Sex(Male)	−0.085	[−0.374, 0.204]	0.554
SCAT change × Sex(Male)	0.0005	[−0.0008, 0.0019]	0.448
Observations: 33			
*R* ^2^ = 0.023, adjusted *R* ^2^ = −0.078			
Model fit: *F*(3, 29) = 0.23, *p* = 0.878			

*Note:* Each model was fitted within the heading group only (*N* = 33) with change in SCAT‐6 total score, sex, and their interaction as predictors; no age term was included, so the residual degrees of freedom are 29. Sex was entered with sum‐to‐zero (effect) coding, so the sex coefficient is the deviation of males from the grand mean rather than a simple male‐minus‐female difference. Intervals are 95% confidence intervals.

Similar regression analyses predicting changes in Composite Error from SCAT‐6 change and sex (including their interaction) did not yield significant main effects or interactions (*F*(3, 29) = 0.78, *p* = 0.516, Adjusted *R* ^2^ = −0.021; all predictors *p* ≥ 0.210). For Composite Reaction Time change, the model was not significant (*F*(3, 29) = 0.23, *p* = 0.878, Adjusted *R* ^2^ = −0.078; all predictors *p* ≥ 0.448) (Table 3).

## 4 Discussion

In this study, a thrown‐to controlled heading drill evoked increased symptom severity, but no appreciable change to cognition, irrespective of participant sex. The main findings of this study are outlined below.

### 4.1 The Effect of Football Headers on Reaction Time

The lack of change in RT and error rate post‐heading are in line with previous reviews (Kontos et al. 2017; McCunn et al. 2021). A similar study design conducted by Di Virgilio and colleagues also reported no effect of heading on RT (Di Virgilio et al. 2016). The persistent null effect of thrown‐to headers on cognitive function RTs may be alternatively explained by variables that transiently enhance immediate cognitive performance, masking the negative impact of headers that could present later. For example, headers may temporarily heighten alertness through a visual looming effect, whereby a fast‐approaching stimulus triggers a sensorimotor response (Maier et al. 2008; Field and Wann 2005; Cléry et al. 2015). Results may be alternatively explained via acute neurometabolic responses to head impacts (i.e., hyperglycolysis), which can obscure early deficits before subsequent hypoglycolysis impairs function (Barkhoudarian et al. 2011). These mechanisms underscore the need for future studies to assess cognition both immediately and at delayed time points post heading. Previous research has also demonstrated a marked decline in cognitive performance immediately after 20 headers (Ashton et al. 2021; Parr et al. 2023). It may be that the sub‐concussive effects of the drill employed by these studies might accumulate to negatively influence cognition beyond that which is explored here (i.e., 10 thrown‐to controlled headers). Furthermore, despite Di Virgilio and colleagues' null findings for RT, the authors demonstrate detriments to the corticomotor silent period (i.e., activity of inhibitory interneurons), spatial working memory, and paired associative learning (Di Virgilio et al. 2016). This suggests that heading differentially alters several cognitive domains. These hypotheses, however, cannot be supported or refuted by the protocols used here.

Future studies which investigate several cognitive domains, as well as both the immediate and persistent cortical and neurometabolic responses to head injury, sub concussive head impacts, and headers that present stronger linear and rotational forces such as those that characterise in‐game scenarios, should be conducted.

### 4.2 Acute Heading Exacerbates Symptom Severity

The results revealed higher symptom severity scores in the heading group compared to the control. These results are in line with several studies indicating that athletes experience a significant increase in symptoms after RHIs (Haran et al. 2012; Schmitt et al. 2004), and these have been suggested to relate to circulating biomolecules and cerebral autoregulation. Wallace and colleagues (Wallace et al. 2018) found that after completing a bout of 40 headers, participants demonstrated increased symptomology and concomitant, elevated serum concentrations of neurofilament light (NF‐L; a biomarker which assesses neuronal injury). The increased NF‐L concentrations persisted for up to 1‐month post‐heading and provide evidence for the potential long‐term effects of a series of headers. Moreover, Smirl and colleagues assessed the effects of 40 headers on symptom severity and dynamic cerebral autoregulation (Smirl et al. 2022). Results demonstrated that participants reported an increase in concussion‐like symptoms and a disruption to autoregulation. It must be acknowledged that these studies involved 40 headers from a further distance (35 m) and at greater incoming velocities (i.e., 77.5 ± 3.7 km/h) than those utilised here. The methodology employed in our study also does not allow to directly assess the mechanisms by which symptomology is altered by a bout of heading. Future research which includes measures of autoregulation and/or circulating biomarkers would shed light on this issue.

### 4.3 The Lack of Sex Differences After a Series of Headers

This study found no differences in RT changes between males and females, a finding which aligns with those produced in previous works (Kontos et al. 2011; Rieder and Jansen 2011). Recent data from collegiate athletes with equivalent access to sports medicine may also support this, showing broadly similar concussion incidence and recovery trajectories between sexes, with the main differences seen in acute symptom burden and vestibular‐ocular findings (Caccese et al. 2024). However, due to the limited body of literature on this topic the findings of the present study must also be viewed in parallel with evidence indicating that females experience higher concussion rates (Dick 2009) and worsened symptom severity compared to males. Previous studies have shown decrements to RT, visual motor‐speed (Chiang et al. 2009), visual memory (Covassin et al. 2007), and vestibular ocular deficits (Clair et al. 2020; Sufrinko et al. 2017; Neidecker et al. 2017). Female players are also shown to experience higher peak impact speeds compared to males (Caccese et al. 2018; Bretzin et al. 2017); a result which has been attributed to weaker flexor and extensor muscles and smaller head mass (Tierney et al. 2008).

In explaining our null findings, we note that the effects of the RHIs observed might not be evident immediately after heading or might require a greater impact load. Rather, it may be that a greater accumulation of heading impacts can explain the sex effects observed elsewhere. This dose‐response effect of greater heading volume and greater cognitive impairment is supported by several retrospective analyses (Macnab et al. 2023; Espahbodi et al. 2023). For example, Espahbodi et al. reported that those with a heading frequency of 15 times per match/training session, possessed more than a three‐fold increased risk of cognitive impairment compared with those with heading frequencies of 0–5 times (Espahbodi et al. 2023). The authors, however, only involved a male sample, and did not compare the effects of a similar dose of heading for females.

### 4.4 Study Limitations

Our study is limited in several methodological aspects. First, group allocation was non‐randomised and based on participant availability rather than random assignment, which may have introduced selection bias. Future studies should therefore randomly allocate participants to help mitigate this bias. Second, the sample comprised recreational and mixed‐ability players rather than elite athletes, which may limit generalisability to professional populations. Third, our study only involved 10 headers. When compared to other studies which involved between 20 and 40 headers, it may be that the null findings observed here were due to an insufficient header burden. Fourth, participants were instructed to complete direct, forward‐facing headers, in quick succession, from a distance of 5 m. This protocol does not capture the variability seen within training or matches. Footballers are often required to head a ball from variable distances and velocities, with differing intervals between headers and in both rotational and direct directions. The inclusion of these types of headers in subsequent work will provide a more complete understanding of the relationship between headers, cognition, and sex; however, standardisation may be more difficult to achieve. Fifth, our study did not employ any means by which the underlying mechanisms of repetitive headers might be quantified. Indeed, future work should employ some measures to assess cortical excitability, circulating blood biomarkers, functional neural‐connectivity or cerebral autoregulation. Sixth, the control group completed a seated rest period rather than an activity of comparable exertion, such as simulating a heading action without ball to head contact, introducing fatigue as a potential compound. Finally, the sex comparisons were statistically underpowered. The absence of significant sex differences should therefore not be read as evidence that none exist, and larger, adequately powered studies are needed to draw firmer conclusions regarding sex‐specific effects of heading.

## 5 Conclusion

This study showed that a controlled header drill had no clear effect on RT, but did elicit a significant increase in SCAT‐6 symptoms, and no sex differences were observed. These findings suggest the importance of considering player‐reported symptoms when developing heading guidelines, as well as providing the groundwork for further research using stronger, match‐representative headers. Studies should also assess both immediate and longer‐term effects across multiple cognitive domains to better describe potential sex‐related differences in cognitive outcomes. This study represents an initial step towards better understanding how repetitive heading might influence short‐ and long‐term cognitive effects for both male and female footballers.

## Funding

The authors have nothing to report.

## Ethics Statement

All participants provided informed written consent of a protocol approved by the University College London Research Ethics Committee (13985.007) prior to undertaking any study activity. These activities aligned with the most recent iteration of the Declaration of Helsinki.

## Conflicts of Interest

The authors declare no conflicts of interest.

## Supporting information


Supporting Information S1

## Data Availability

The datasets generated and analysed during the current study are available from the corresponding author on reasonable request.
