# Supplementary material for: Short Term Effects of Football Headers on Reaction Time and Symptomology in Males and Females
Source: Eur J Sport Sci. 2026 Oct 7;26(11):e70251. doi: 10.1002/ejsc.70251 (PMC13644708; doi:10.1002/ejsc.70251)
Supplement: Supplementary file 1 [file EJSC-26-e70251-s001.docx]

**Supplementary Material**

| **(A)**   | **(B)**   |
| --- | --- |

**Supplementary Figure S1.** Modified Sport Concussion Assessment Tool, 6th edition (SCAT-6) symptom scale as administered via the Gorilla platform. Participants rated each of 15 symptoms for how they felt “right now” by moving a continuous visual analogue slider anchored at “Not at all” (0) and “Extremely” (100); the numerical value was displayed above the handle as it was moved. (A) Items 1–9: dizziness, increased pressure in the head, neck pain, headache, not feeling right, feeling slowed down, feeling more emotional than usual, feeling in a fog, and blurred vision. (B) Items 10–15: sensitivity to light or noise, balance problems, confusion and difficulty concentrating, drowsiness, irritability, and feeling nervous or anxious. Total SCAT score was calculated as the sum of all 15 item scores (range 0–1500), with higher scores indicating greater symptom severity. The scale was administered immediately before and immediately after the heading drill (heading group) or the 5-minute seated rest period (control group). Sliders are shown at their default starting position.

**Supplementary Table S1.** Wording of the 15 modified SCAT-6 symptom items, in the order presented to participants, with the corresponding variable labels used in the analysis.

| **No.** | **Item as presented to participants** | **Variable label** |
| --- | --- | --- |
| 1 | “Do you feel dizzy right now?” | *Dizziness* |
| 2 | “Do you feel any increased pressure in your head right now?” | *Pressure in head* |
| 3 | “Do you feel any neck pain right now?” | *Neck pain* |
| 4 | “Do you have a headache right now?” | *Headache* |
| 5 | “Do you not feel right right now?” | *Not feeling right* |
| 6 | “Do you feel slowed down right now?” | *Feeling slowed down* |
| 7 | “Do you feel more emotional than usual right now?” | *More emotional* |
| 8 | “Do you feel like you are in a fog right now?” | *Feeling in a fog* |
| 9 | “Are you experiencing any blurred vision right now?” | *Blurred vision* |
| 10 | “Are you experiencing any sensitivity to light or noise right now?” | *Sensitivity to light or noise* |
| 11 | “Are you experiencing any balance problems right now?” | *Balance problems* |
| 12 | “Are you experiencing any confusion and difficulty concentrating right now?” | *Confusion / difficulty concentrating* |
| 13 | “Are you experiencing any drowsiness right now?” | *Drowsiness* |
| 14 | “Are you experiencing any irritability right now?” | *Irritability* |
| 15 | “Are you feeling nervous or anxious right now?” | *Nervousness / anxiety* |

*Note.* All items were scored on a 0–100 visual analogue scale (0 = “Not at all”, 100 = “Extremely”). This differs from the published SCAT-6 symptom scale, which uses a 0–6 Likert response format (maximum total 132). Each slider was presented at a default starting position of 50, and participants were required to register a response for every item.
